# Emission Characteristics of Particulate Matter, Volatile Organic Compounds, and Trace Elements from the Combustion of Coals in Mongolia

**DOI:** 10.3390/ijerph15081706

**Published:** 2018-08-09

**Authors:** Mona Loraine M. Barabad, Wonseok Jung, Michael E. Versoza, Minjeong Kim, Sangwon Ko, Duckshin Park, Kiyoung Lee

**Affiliations:** 1 Transportation Environmental Research Team, Korea Railroad Research Institute, Uiwang City 16105, Korea; misseuphoric@krri.re.kr (M.L.M.B.); worship611@krri.re.kr (W.J.); mikeverz23@krri.re.kr (M.E.V.); mjkim88@krri.re.kr (M.K.); sko@krri.re.kr (S.K.); 2 Railway System Engineering, University of Science and Technology, 217 Gajeong-ro, Yuseong-gu, Daejeon 34113, Korea; 3 Department of Environmental Health and Institute of Health Sciences and Environment, Graduate School of Public Health, Seoul National University, 1 Gwanak-ro, 1 Gwanak-gu, Seoul 08826, Korea

**Keywords:** coal, combustion, particulate matter (PM), volatile organic compounds (VOCs), emission

## Abstract

This study characterized emissions of particulate matter (PM), volatile organic compounds (VOCs), heavy metals, and anions from Mongolian bituminous coals in a controlled heating experiment. Three coal samples from Alag Tolgoi (coal 1), Baganuur (coal 2), and Nalaikh (coal 3) were combusted at a constant heat flux of 50 kW/m^2^ using a dual-cone calorimeter. The coal samples were commonly used in ger district of Ulaanbaatar, Mongolia. PM_10_ emission factors were 1122.9 ± 526.2, 958.1 ± 584.0, and 472.0 ± 57.1 mg/kg for coal samples 1, 2, and 3, respectively. PM with a diameter of 0.35–0.45 µm was dominant and accounted for 41, 34, and 48% of the total PM for coal samples 1, 2, and 3, respectively. The emissions of PM and VOC from coals commonly used in Ulaanbaatar, Mongolia were significant enough to cause extremely high levels of indoor and outdoor air pollution.

## 1. Introduction

In many underdeveloped countries, solid fuels (such as wood, animal dung, crop wastes, and coal) are used for heating and cooking in residences. According to the World Health Organization (WHO), around 3 billion people used solid fuels for heating and cooking using open fires and simple stoves [1]. However, due to the incomplete combustion of these fuels, significant amounts of harmful pollutants could be emitted to the environment and affect human health. Indoor air pollution from solid fuels could be the cause of significant mortality and morbidity of women and children worldwide [2]. The use of solid fuels was associated with health problems, such as chronic obstructive pulmonary disease (COPD) [3] and lung cancer [4], which tended to occur in women who were exposed to pollutants when cooking with solid fuels [5]. Conditions such as tuberculosis [6], asthma [7], cardiovascular disease [8], and cataracts [9,10] were associated with exposure to emissions from biomass fuel combustion. Health issues, such as low birthweight [11], inadequate growth development [12], and acute lower respiratory infections [13], were also reported among individuals exposed to such emissions.

Coal has been used as a major household fuel in many developing countries. Although the use of coal was discouraged or banned in many Chinese cities, about 42% of households still used it for cooking and 35.6% of households used it for heating [14]. Coal was also used in homes in various South Asian countries, including India, where it was widely used for cooking [15], while coal and charcoal were commonly burned in homes in Bangladesh, Sri Lanka, Nepal, and some parts of South Africa. Most of these households used unprocessed coals, although the World Health Organization (WHO) guideline strongly recommended that unprocessed coals should not be used as a household fuel [16].

Indoor air pollution from coal combustion was designated as a group I carcinogen by the International Agency for Research on Cancer (IARC). Combustion of coal produces various air pollutants, including particulate matter (PM) and volatile organic compounds (VOCs). PM from coal combustion (meat charbroiling) was mainly fine and ultrafine (mass distribution peaked between 0.1 and 0.2 µm particle diameter) [17]. Fresh coal smoke contained a large number of ultrafine particles, which condense as they age. Coal combustion is associated with high levels of indoor exposure to PM and polyaromatic hydrocarbons (PAHs) [18,19] in China. When coal was burned in households, carcinogenic substances such as benzene, styrene. ethylene, acetylene, and propane were measured [20]. Coal contained high levels of intrinsic toxicants including fluorine (F), arsenic (As), lead (Pb), selenium (Se), and mercury (Hg). These heavy metals were associated with adverse health effects. In certain parts of China, health problems such as arsenosis and fluorosis were observed as a result of the use of coal [21]. Several studies also have reported of effective gaseous emission treatment technique for coal combustion, a combination of fly-ash products (FAP) and Zeolite 13X was described to enhance the removal of Toluene [22]. In addition, coal combustion is one of the greatest sources of anthropogenic mercury (Hg) [23], hence, mercury control techniques have been addressed, activated carbon is effectively applied for the adsorption of mercury, fly ash may also promotes Hg emission control with using fabric filters [24,25].

For risk assessment of the coal combustion, the study identified the possible sources of pollutants from the combustion process, on the assumption that the products could also be found in an actual indoor setting. Hence, the causes of these practices should be recognized for background study. With regards to environmental and health aspects, this work considered the potential threats from the exposure of the harmful products emitted and produced from the coal combusted. The purpose of this study was to determine the size distribution of PM and the specific VOCs emitted from the combustion of selected coals used in Mongolia. Trace amounts of heavy metals and anions in PM from coal combustion were also determined. The study characterized the air pollution emissions of coal combustion in controlled experiments.

## 2. Experiments

This study used unprocessed bituminous coals from different mines in Mongolia. Three different coals were collected from the three main coal supply chains in Ulaanbaatar, Mongolia; Alag Tolgoi (coal 1), Baganuur (coal 2), and Nalaikh (coal 3). Table 1 shows an elemental analysis of these coals. Coal 1 had a higher carbon content (63.5%) than coal 2 (54.0%) and coal 3 (58.6%). However, coal 2 had a higher content of both oxygen (20.0%) and ash (20.9%) compared to coal 1 (17.0% and 13.7%, respectively) and coal 3 (18.6% and 16.7%, respectively). It should be noted that these coals did not represent the Mongolian coal supply as a whole. 

**Figure 1:**
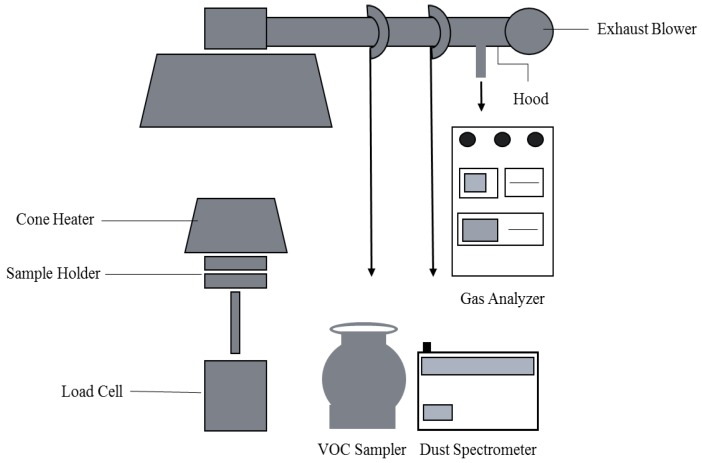
Schematic diagram of the cone calorimeter used in the coal combustion experiment.

A dual-cone calorimeter (Fire Testing Technology; East Grinstead, UK) was used to observe the generation of PM and VOCs from coal samples. Figure 1 shows a schematic diagram of the experimental system. The dual cone calorimeter consisted of a conical heater, temperature controller, split shutter mechanism, sample holder, load cell, spark ignition, circuit exhaust system, gas sampling, heat flux meter, oxygen analyzer, smoke obscuration system, calibration burner, data acquisition and analysis system, nondispersive infrared (NDIR) gas analyzer for CO and CO_2_, and protective screens. The built-in analyzer measured the heat release rate (HRR, kW/m^2^), time of ignition [TTL (s)], critical ignition flux, mass loss rates, total smoke release rate (TSR), time to flameout [TTF (s)], effective heat of combustion, oxygen consumption rate (OCR), and release rates of toxic gas (e.g., CO_2_ concentration %).

The truncated conical heater was rated 5000 W at 230 V, with a maximum heat output of 100 kW/m^2^. For this analysis, the temperature was only set at 50 kW/m^2^, with an approved temperature of 735 °C from the International Organization for Standardization (ISO 5660-1). The samples were placed in the specimen holder with dimensions of 100 mm (width) × 100 mm (length) × 50 mm (depth). The exhaust system, which comprised the hood, gas sampling ring probe, exhaust fan, and an orifice plate flow measurement was operated normally at a nominal 24 L/s. The NDIR gas analyzers (CO and CO_2_) were calibrated using standard gases. The oxygen analyzer tested the oxygen level when it was calibrated using the air collected (connected from a port outside). 

A 16-channel dust spectrometer (1.108; Grimm Technologies Inc., Ainring, Germany) was used to measure the size distribution of particles, with a flow rate of 1.6 L/min. The sampling inlet of the monitor was placed inside the duct of the cone calorimeter. The measurement was adjusted by the weight of dust collected inside the spectrometer. The aerosol particle is detected in the optical measuring cell and allocated to a defined particle size, based on the intensity of the scattering light signal and the device allows to count and analyze the sample collected (Grimm-Aerosol, Ainring, Germany).

A 6-L silicon canister (Restek Co., Bellefonter, PA, USA), with an internal surface and valve coated with inert silica to prevent the adsorption of VOCs, was used to collect air samples and control the flow rate. The sampling inlet was aligned with a dust spectrometer. VOC sampling was conducted for 1 min during combustion experiments. The collected samples were pre-concentrated in the lab and analyzed by gas chromatography/mass spectrometry (GC/MS). The GC/MS system determined the levels of 57 chemicals designated by the United States Environmental Protection Agency (US EPA) as key substances for the consideration of toxicity due to their potential carcinogenic and mutagenic properties. 

During the combustion, the total suspended particles inside the duct of the cone calorimeter were collected on a quartz microfiber filter paper (203 × 254 mm). The filter paper was cut in half, digested with concentrated HNO_3_ and HCl, and heated in a microwave. The digested samples were filtered, and the filtrates were collected and used to detect trace elements in the coal smoke samples using inductive coupled plasma optical emission spectrometry (ICP-EOS, 720 Series; Agilent Technologies, Santa Clara, CA, USA). The other half of the filter paper was cut into smaller pieces, dissolved with distilled water, and sonicated for conversion into a liquid form. This was then used for distinguishing anions using ion chromatography (IC) (Metrohm 761 Compact IC, Herisau, Switzerland). A Multi Anion Standard 1 for IC (Sigma-Aldrich Production, Buchs/Switzerland) was used for ion chromatography calibration and in accordance to ISO Guide 31. The test was conducted for two (2) weeks with 60 samples, moreover Anion values were determined and analyzed.

Emission factors for PM and VOCs for the different coal samples were calculated by the measurement of flue gas volume and the mass concentration of pollutants using Equation (1). 

Emission factors were expressed in milligrams of pollutants per kilogram of combusted coal sample.

(1) $$\mathrm{EF}\;\left(\mathrm{mg}/\mathrm{kg}\right)=\frac{\mathrm{concentration}\;\mathrm{of}\;\mathrm{pollutants}\;(\mathrm{mg}/m^{3})\times \mathrm{flow}\;\mathrm{rate}\;(m^{3}/\min)\times \mathrm{sampling}\;\mathrm{time}\;\left(\min\right)}{\mathrm{weight}\;\mathrm{of}\;\mathrm{burned}\;\mathrm{coal}\;\mathrm{sample}\;\left(\mathrm{kg}\right)}$$

Concentrations of pollutants were determined using a dust spectrometer for PM and GC/MS for VOCs. Flow rate, total sampling time, and the weight of burnt material were recorded automatically by the cone calorimeter. The coal specimens were burned three times each. SPSS software (ver. 12; SPSS, Inc.) was used to statistically analyze the data. 

## 3. Results

Table 2 shows the results of the coal combustion experiments. The average initial mass of the coal samples was 10.0 g. At a heat flux of 50 kW/m^2^, the samples lost approximately 92% of their total mass, while 7–8% remained after the combustion of all samples. The time of ignition varied among the different samples. The ignition time of coal 1 was the fastest, while coal 2 was ignited slowly. There was a similar pattern for the flameout timings. The recorded times were 165.0 ± 52.4, 136 ± 20.3, and 154.7 ± 33.3 s for coal 1, coal 2, and coal 3, respectively. The peak heat release rate (pk-HRR) of coal 1 was twice as high as that of coal 2 and coal 3. The pk-HRR was 101.6 ± 16.73 for coal 1, 54.7 ± 11.1 for coal 2, and 50.7 ± 21.2 kW/m^2^ for coal 3. The CO_2_ emission factor was lowest for coal 1, with a value of 1151.2 ± 1028.3 mg/kg, while coal 3 had the highest emission factor of 2519.7 ± 2245.9 mg/kg, followed by coal 2 with a value of 2032.44 ± 800.2 mg/kg. The CO emission factors for coals 2 and 3 were similar, and coal 1 had the lowest emission factor of 154.1 ± 153.5 mg/kg.

**Figure 2:**
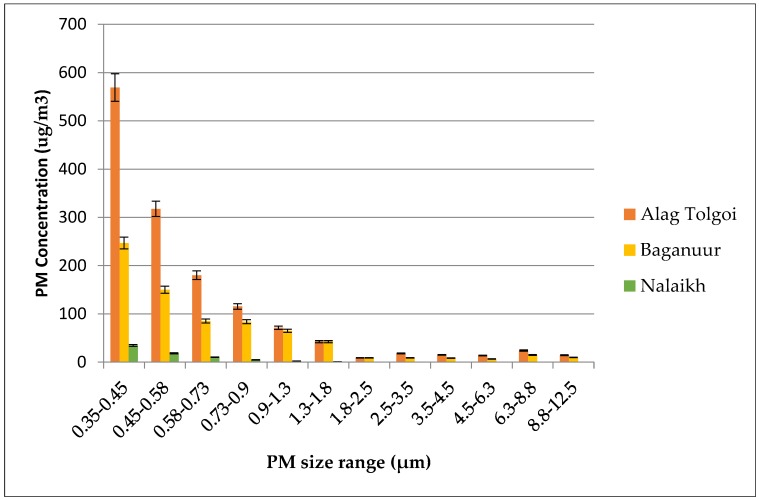
Size distribution of particulate matter (PM) from different types of coal.

The particle size distribution was different between coal samples, as shown in Figure 2. The greatest percentage of particles was located in the size class between 0.35 and 0.45 µm. Particles with a diameter between 0.35 and 0.45 µm comprised 41%, 34%, and 48% of the PM_10_ mass concentrations from coals 1, 2, and 3, respectively. The highest concentration of particles in the size class between 0.35 and 0.45 µm was from the combustion of coal 1, which was 2.3 and 16.5 times higher than that in coals 2 and 3, respectively. Following the combustion of coal 1, particles with a diameter between 0.45–0.58 µm and 0.58–0.73 µm accounted for 22.9% and 13.0% of the total particles (or PM_10_), respectively. PM_2.5_ concentrations were 93.87, 93.41, and 99.85% of the PM_10_ concentration following the combustion of coals 1, 2, and 3, respectively. PM_1.0_/PM_10_ ratios were 90.19%, 86.41%, and 98.14% following the combustion of coals 1, 2, and 3, respectively. The similarity of the PM_1.0_/PM_10_ ratio and the PM_2.5_/PM_10_ ratios indicated that the majority of PM emitted during combustion was PM_1.0_ at the high heat fluxes.

The emission factors of PM_2.5_ and PM_10_ varied among the different coal types, as shown in Table 3. The PM_10_ emission factors were 1122.9 ± 135.4, 958.1 ± 584.0, and 472.0 ± 57.1 mg/kg for coals 1, 2, and 3, respectively. The PM_2.5_ emission factors were 1043.5 ± 458.0, 892.7 ± 545.5, and 471.5 ± 57.0 mg/kg for coals 1, 2, and 3, respectively whereas PM_1.0_ emission factors were 1002.5 ± 422.1, 822.8 ± 504.6, and 461.2 ± 56.0 mg/kg for coals 1, 2, and 3, respectively.

Table 4 presents the concentration and emission factors of VOCs collected from the combustion of coal samples. A total of 19 VOCs was detected from coal 2 emissions, while a total of 17 VOCs were identified from coals 1 and 3. Acetone and isopropyl alcohol were present at concentrations higher than 100 μg/m^3^ in emissions from all coals. In the emissions from coal 1, benzene and toluene concentrations were higher than 100 μg/m^3^, whereas the emissions from coal 2, methylethyl ketone, tetra hydrofuran, benzene, and toluene concentrations were higher than 100 μg/m^3^. As for the emissions from coal 3, methylethyl ketone and tetra hydrofuran concentrations were higher than 100 μg/m^3^. 

Emission factors for the majority of the VOCs differ among the coal samples. Benzene emission factors were 15%, 16%, and 9% of the total VOC emission factors for coals 1, 2, and 3, respectively. Acetone emission factors were 12%, 15%, and 18% of the total VOC emission factors for coals 1, 2, and 3, respectively. Toluene emission factors were 14%, 14%, and 11% of the total VOC emission factors for coals 1, 2, and 3, respectively. Methyl ethyl ketone emission factors were 9%, 11%, and 17% of the total VOC emission factors for coals 1, 2, and 3, respectively. Tetra hydrofuran emission factors were 11%, 12%, and 13% of the total VOC emission factors for coals 1, 2, and 3, respectively. Xylene emission factors were 11%, 9%, and 9% of the total VOC emission factors for coals 1, 2, and 3, respectively.

The results of the analysis of trace metals in emissions from the combustion of the three coal samples are shown in Table 5. Numerous elements were detected in emissions from all of the coal samples, but silver (Ag), cadmium (Cd; for coal 1 and coal 3), cobalt (Co; for coal 2), In, Pb (for coal 3) and titanium (Ti) were not detected. The concentrations of boron (B) and Ca were notably higher than the other trace elements in the emissions from all coal samples. The emission factors of Ca were 2774.0, 3386.7, and 4057.0 mg/kg for coals 1, 2, and 3, respectively, and the emission factors of B were 1339.1, 981.9 and 797.2 mg/kg for coals 1, 2, and 3, respectively. In addition, emission factors of iron (Fe) were 873.5, 1206.1 and 1140.6 mg/kg for coals 1, 2, and 3, respectively. Toxic metals such as Pb, Cd, chromium (Cr), copper (Cu), nickel (Ni), and zinc (Zn) were detected at low concentrations.

Table 6 shows the concentration and emission factors of anions from the IC analysis of the coal combustion products. The majority of the anions, including $F^{-}$, ${\mathrm{Cl}}^{-}$, NO_3_^−^, and SO_4_^2−^ were identified, whereas ${\mathrm{Br}}^{-}$ and PO_4_^3−^ were undetected. In all samples, the anion with the highest concentration was SO_4_^2−^, followed by NO_3_^−^, $F^{-},$ and ${\mathrm{Cl}}^{-}$. The emission factors of SO_4_^2−^ were 6937.0, 5409.0, and 2430.0 mg/kg for coal samples 1, 2, and 3, respectively. In contrast to the emissions from the combustion of coal 1 having the highest trace metal concentrations, the combustion of coal 3 released the highest concentration of anions.

## 4. Discussion

The PM and VOC emission factors for unprocessed Mongolian coals were determined from controlled heating experiments. A cone calorimeter was used for the combustion of the coal, with a constant heat flux of 50 kW/m^2^. A heat flux of 50 kW/m^2^ was selected because it is relatively close to the combustion conditions of coal. The results from the experiment may therefore only be applicable to the combustion period.

The emission characteristics during ignition may be different from those during the time of combustion. The PM_10_ emission factor from the combustion of cow dung in the ignition period was 13–80 times higher than in the combustion period [26]. Similarly, the PM emission factor from the combustion of coal may be higher in the ignition period. There was an initial attempt to apply two different heat fluxes of 15 and 25 kW/m^2^, however, these heat fluxes were too low to generate fire and to achieve complete combustion of the coal samples. Coal is often ignited with the combustion of another organic material, such as paper or wood, experimental results at a lower heat flux or temperature may not be applicable to field conditions. Because the combustion stage is the period in which the majority of heating and cooking with coal occurs, the emission factor determined in this study could represent field conditions. 

The CO_2_ emission factors results were distinguished among the different coal samples. Coal 1 had the lowest CO_2_ emission factor. Although coal 1 had a slightly higher carbon content, its peak HRR was twice as high as the other coals. Peak HRR is an indicator of energy content. Because CO_2_ emissions are affected by the carbon content, and are the inverse of the energy content, the lower CO_2_ emission factor of coal 1 is likely to be due to its high energy content. The CO_2_ emission factors of coals 2 and 3 were much higher than the reported CO_2_ emission factor of cow dung combustion at 50 kW/m^2^ having the value of 1407.7 ± 138.2 mg/kg [26]. Moreover, the CO emission factors of coal combustion were also much higher than the reported value for cow dung combustion. 

The PM emission factors were significantly different among the different coal samples. The PM_10_ emission factor of coal 1 was about 2.4 times higher than that of coal 3 and the PM_10_ emission factor of coal 2 was about two times higher than that of coal 3. Assuming similar burning conditions, the use of coal 1 and 2 could generate more PM pollution. The emission characterization results were difficult to directly compare to field concentrations. The PM_10_ emission factors of coal combustion at 50 kW/m^2^ were 1122, 958, and 472 mg/kg for coals 1, 2, and 3, respectively. If the typical amount of coal used each day is 26.4 kg/day [27], 12,460–29,647 mg of PM_10_ could therefore be generated. With a volume of 42 m^3^ in a traditional Ger and with an assumption of a 10% leakage rate from stove to Ger, PM_10_ concentrations from coals 1, 2, and 3 could theoretically reach levels of 70, 60, and 30 mg/m^3^, respectively, without ventilation. The particle size distribution varied among the different coal samples, even with combustions at the same temperature. Most of the particulate mass emitted from bituminous coal combustion consisted of submicron particles. The predominant particle size was about 0.4 μm. The size distributions of the average emitted particles were broader than the atmospheric size distributions [28]. The data indicated that smaller particle sizes were abundant and could potentially affect human health. According to a previous study, fine and ultrafine particles (<1 µm in diameter) are emitted from coal combustion [29]. Combustion-generated particles have different chemical compositions, and because particle size determines how deep the particles can penetrate the respiratory tract, determining size distributions is critical in determining health impacts [30].

The VOCs concentrations in emissions from coal combustion were also measured. The number of VOCs detected varied among the coal samples. Benzene, toluene, acetone, and isopropyl alcohol were detected at concentrations higher than 100 μg/m^3^ for coal 1. Benzene, acetone, toluene, tetra hydrofuran, methyl ethyl ketone, and isopropyl alcohol were detected at concentrations higher than 100 μg/m^3^ for coal 2. Acetone, methyl ethyl ketone, tetra hydrofuran, and isopropyl alcohol were detected at concentrations higher than 100 μg/m^3^ for coal 3. Coal 2 contained more VOCs than the other types of coal and they were also present at higher concentrations. 

The emission factors of the VOCs among the coal samples also varied from the results. The emissions from the combustion of coal 2 contained six compounds with an emission factor higher than 100 mg/kg: benzene, acetone, toluene, tetra hydrofuran, methyl ethyl ketone, and isopropyl alcohol. The emissions from the combustion of coal 3 contained six compounds with an emission factor higher than 100 mg/kg: acetone, methyl ethyl ketone, and tetra hydrofuran. The emissions from the combustion of coal 1 contained two compounds with an emission factor higher than 100 mg/kg: benzene and toluene. Different emission factors from the combustion of different types of coal have also been reported in another study [31]. The emission factors of benzene and toluene were reported to range between 7–27 and 12–40 mg/kg for six different types of coal. The coals were from South Africa and Spain. The emission factors were generally lower than the emission factors of the Mongolian coal. 

The high emission factor of calcium (Ca) indicated the presence of calcite in coals. Coal often contains pyrite (Fe), alkaline materials, and Ca. Calcite is the dominant Ca-bearing mineral in bituminous coal samples [32,33]. Carboxyl-bound Ca and minor phases containing Ca–Sr–Al–P were found to be the major Ca-rich forms of a bituminous coal sample (Eagle Butte coal) [34]. The Inductive Coupled Plasma—Optical Emission Spectrometry (ICP-OES) analysis results showed the presence of toxic materials in the emissions from coal combustion. The US EPA identified 11 elements (As, beryllium (Be), Cd, Co, Cr, Hg, manganese (Mn), Ni, Pb, antimony (Sb), and Se) as being air pollutants hazardous to the environment and human health [35]. Moreover, Cd, Ni, and Pb are of prime environmental concern to the European Union and the Canadian Environmental Protection Agency because there were notable emission factors of Mn, Co, Pb, Cr, and Ni from the combustion of the coals tested here, the results suggest potential health risks. The analytical results did not distinguish between Cr (IV) and Cr (IIIVa). When Cr is heated at high temperatures, Cr (VI) is converted to Cr (III) [36]. Although Cr (III) is less toxic, it can still pose a health risk. More detailed analysis of the speciation of Cr is required in future coal combustion studies. 

The IC results revealed the presence of SO_4_^2-^ and NO_3_^-^, along with $F^{-}$ and ${\mathrm{Cl}}^{-}$ in the emissions from the coal combustion, although ${\mathrm{Br}}^{-}$ and PO_4_^3−^ were absent. Moreover, the uncertainty was calculated based on the Eurachem/Citac Guide, and showed a 95% confidence level. Concentrations of SO_4_^2−^ and NO_3_^−^ from residential coal combustion emissions have been reported to be four times higher than emissions from residential wood combustion [37]. The emission of ${\mathrm{Cl}}^{-}$ from residential wood combustion is 55% higher than that from residual coal combustion. 

## 5. Conclusions

The emission factors of various air pollutants from coal combustion were determined in a controlled laboratory experiment. Although controlled heating experiments can provide accurate emission factor data, the results might not be directly applicable to field conditions. In Gers, burning conditions, the type of stove, the type of coal, and the actual amount of coal used may affect the emission of air pollutants. Ultrafine particles (<PM_1_) and toxic compounds such as benzene and toluene were found to be most significant, heavy metals were also present from the combustion process, exposure from these products are a risk to households. In addition, the characteristics of PM and VOCs in the field may change after emission. Coal combustion is a continuous practice in several developing countries, several alternative methods have been introduced, however, public awareness is needed to prevent and/or alleviate health and environmental problems caused from biomass and coal burning.

## Figures and Tables

**Table 1:** Elemental analysis of coal samples.

Sample Name	Nitrogen (%)	Carbon (%)	Hydrogen (%)	Sulphur (%)	Oxygen (%)
Alag Tolgoi (Coal 1)	1.0	63.5	4.2	0.5	17.0
Baganuur (Coal 2)	0.7	54.0	4.0	0.4	20.0
Nalaikh (Coal 3)	1.2	58.6	4.6	0.2	18.6

**Table 2:** Experimental results for the combustion of different coals.

		Alag Tolgoi (Coal 1)	Baganuur (Coal 2)	Nalaikh (Coal 3)
Mass (g)	Initial	10.0 ± 0.0	10.0 ± 0.0	10.0 ± 0.0
	Loss	9.3 ± 0.1	9.2 ± 0.1	9.3 ± 0.1
	Remaining	0.7 ± 0.1	0.8 ± 0.1	0.7 ± 0.1
Time (s)	Ignition	37.3 ± 7.8	80.7 ± 8.7	64.3 ± 10.7
	Flameout	165 ± 52.4	136 ± 20.3	154.7 ± 33.3
Heat (kW/m^2^)	THR *	18.4 ± 1.9	17 ± 4.3	13.1 ± 1.5
	Peak HRR	101.6 ± 16.7	54.7 ± 11.1	50.7 ± 21.2
CO_2_ emission factor (mg/kg)		1151.2 ± 1028.3	2032.4 ± 800.2	2519.7 ± 2245.9
CO emission factor (mg/kg)		154.1 ± 153.5	178.3 ± 87.8	175.6 ± 166.2

* THR: total heat release; HRR: heat release rate.

**Table 3:** PM emission factors (mg/kg) by particle size for the combustion of different coal types.

PM Size (μm)	Alag Tolgoi (Coal 1)	Baganuur (Coal 2)	Nalaikh (Coal 3)
0.35–0.45	455.3 ± 99.7	304.4 ± 197.5	215.5 ± 27.6
0.45–0.58	254.0 ± 127.0	200.3 ± 120.0	118.1 ± 14.6
0.58–0.73	144.0 ± 83.6	114.0 ± 68.1	77.4 ± 8.2
0.73–0.9	92.2 ± 66.9	115.0 ± 67.0	33.3 ± 3.7
0.9–1.3	57.0 ± 44.8	89.3 ± 52.0	16.9 ± 1.8
1.3–1.8	33.9 ± 29.0	58.2 ± 33.9	9.0 ± 0.8
1.8–2.5	7.0 ± 7.0	11.6 ± 7.0	1.4 ± 0.2
2.5–3.5	17.2 ± 14.4	11.6 ± 7.2	0.1 ± 0.1
3.5–4.5	14.0 ± 12.1	10.9 ± 6.6	0.3 ± 0.0
4.5–6.3	12.9 ± 11.0	8.9 ± 5.2	0.0 ± 0.0
6.3–8.8	21.0 ± 19.2	20.4 ± 11.8	0.0 ± 0.0
8.8–12.5	14.4 ± 11.1	13.5 ± 7.8	0.0 ± 0.0
Total	1122.9 ± 526.2	958.1 ± 584.0	472.0 ± 57.1

**Table 4:** Average concentration and emission factors of volatile organic compounds (VOCs) collected released during coal combustion.

Compounds	Concentration (μg/m^3^)			Emission Factor (mg/kg)		
	Alag Tolgoi (Coal 1)	Baganuur (Coal 2)	Nalaikh (Coal 3)	Alag Tolgoi (Coal 1)	Baganuur (Coal 2)	Nalaikh (Coal 3)
Acetone	110.7 ± 21.5	201.9 ± 86.1	197.7 ± 110.6	88.6 ± 17.2	161.5 ± 68.9	158.2 ± 88.5
Isopropyl alcohol	110.1 ± 5.1	129.5 ± 17.8	111.6 ± 33.7	88.1 ± 4.1	103.6 ± 14.2	89.3 ± 26.9
Methyl ethyl ketone	82.6 ± 37.6	157.2 ± 68.2	189.7 ± 168.0	66.1 ± 30.0	125.8 ± 54.6	151.7 ± 134.4
Tetra hydrofuran	98.5 ± 19.3	166.1 ± 6.4	144.8 ± 40.2	78.8 ± 15.4	132.9 ± 5.1	115.8 ± 32.2
Benzene	131.4 ± 34.8	225.3 ± 168.7	99.7 ± 40.2	105.1 ± 27.8	180.2 ± 134.9	79.8 ± 20.7
Toluene	128.4 ± 17.0	190.3 ± 160.2	99.7 ± 25.9	102.7 ± 13.7	152.2 ± 128.2	98.7 ± 58.2
m,p-xylene	61.7 ± 8.5	91.9 ± 75.6	74.8 ± 48.0	49.3 ± 6.8	73.5 ± 60.5	59.8 ± 38.4
Heptane	54.3 ± 4.3	43.0 ± 29.5	28.4 ± 11.7	43.4 ± 3.4	34.4 ± 23.6	22.7 ± 9.4
o-xylene	33.5 ± 5.3	36.7 ± 28.5	27.4 ± 16.4	26.8 ± 4.2	29.4 ± 22.8	21.9 ± 13.1
Ethylbenzene	23.8 ± 2.3	41.9 ± 30.8	24.3 ± 14.5	19.0 ± 1.8	33.5 ± 24.6	19.6 ± 11.6
1,2,4-Trimethylbenzene	28.3 ± 5.7	29.0 ± 14.2	27.0 ± 14.4	22.6 ± 4.6	23.2 ± 11.3	21.6 ± 11.5
4-Ethyltoluene	16.1 ± 3.4	20.3 ± 11.7	17.8 ± 9.7	12.9 ± 2.8	16.2 ± 9.4	14.2 ± 7.8
1,3,5-Trimethylbenzene	11.7 ± 7.6	12.0 ± 4.3	17.1 ± 16.7	9.3 ± 6.1	9.6 ± 3.4	13.7 ± 13.4
Cyclohexane	4.0 ± 0.3	5.1 ± 33.3	3.2 ± 1.3	3.2 ± 0.2	4.1 ± 2.6	2.6 ± 1.0
Styrene	2.7 ± 2.0	22.9 ± 40.0	8.0 ± 6.3	2.1 ± 1.8	18.3 ± 32.0	6.4 ± 5.0
Ethyl acetate	ND	4.1 ± 0.6	1.8 ± 1.0	ND	3.3 ± 0.5	1.4 ± 0.8
Hexane	7.0 ± 4.6	4.9 ± 3.3	ND	5.6 ± 3.7	3.9 ± 2.6	ND
MIBK	ND	1.6 ± 0.2	1.8 ± 1.2	ND	1.3 ± 0.2	1.5 ± 0.9
1,2,4-Trichlorobenzene	0.7 ± 0.2	3.0 ± 1.8	ND	0.6 ± 0.2	2.4 ± 1.4	ND

ND, not detected.

**Table 5:** Average concentration and emission factor of trace metals released during coal combustion.

Elements	Concentration (μg/m^3^)			Emission Factor (mg/kg)		
	Alag Tolgoi (Coal 1)	Baganuur (Coal 2)	Nalaikh (Coal 3)	Alag Tolgoi (Coal 1)	Baganuur (Coal 2)	Nalaikh (Coal 3)
Al	48.6	32	45.2	38.8	25.6	36.2
B	1673.9	1227.3	996.6	1339.1	981.9	797.2
Ba	22.47	16.8	16.9	18.0	13.5	13.5
Bi	2059.9	1247.9	623.9	1647.9	998.3	499.2
Ca	3467.5	4233.4	5071.2	2774.0	3386.7	4057.0
Cd	0	4.6	0	0	3.7	0
Co	9.64	0	7.2	7.7	0	5.8
Cr	1950.3	195.7	204.2	1560.2	156.6	163.3
Cu	49.4	41.6	18.2	39.5	33.3	14.6
Fe	1091.9	1507.6	1425.4	873.5	1206.1	1140.3
Ga	94.1	42.8	88.4	75.3	34.2	70.7
K	421.2	2398. 0	177.1	337.0	1918.4	141.7
Li	34.6	206.4	1.1	27.7	165.1	0.9
Mg	473.3	465.3	766.6	378.6	372.3	613.3
Mn	24.7	18. 0	22.5	19.8	14.4	18.0
Na	772.9	1106.7	1079.4	618.3	885.4	863.6
Ni	184.8	180. 0	144	147.9	144.0	115.2
Pb	525.4	305.1	0	420.3	244.1	0
Sr	32.3	28.7	39.4	25.8	22.9	31.5
Zn	283.4	240.7	542.8	226.8	192.5	434.3

Ag, In, Ti: Not detected.

**Table 6:** Emission factors (mg/kg) of trace anions released during coal combustion.

	$F^{-}$	$Cl^{-}$	$Br^{-}$	NO_3_^−^	PO_4_^3−^	SO_4_^2−^
Alag Tolgoi (coal 1)	67.8	135.7	0.0	484.8	0.0	6937.0
Baganuur (coal 2)	76.5	165.9	0.0	821.6	0.0	5409.0
Nalaikh (coal 3)	103.8	301.6	0.0	876.4	0.0	2430.0

## References

[1] World Health Organization Household Air Pollution and Health, Key Facts. http://www.who.int/mediacentre/factsheets/fs292/en/.

[2] Fullerton D., Bruce N., Gordon S.B. (2008). Indoor air pollution from biomass smoke is a major health concern in the developing world. Trans. R. Soc. Trop. Med. Hyg..

[3] Bruce N., Perez-Padilla R., Albalak R. (2000). Indoor air pollution in developing countries: A major environmental and public health challenge. Bull. World Health Organ..

[4] Hosgood H.D., Wei H., Sapkota A., Choudhury I., Bruce N., Smith K.R., Rothman N., Lan Q. (2011). Household coal use and lung cancer: Systematic review and meta-analysis of case-control studies, with an emphasis on geographic variation. Int. J. Epidemiol..

[5] Desia M.A., Mehta S., Smith K.R. Indoor Smoke from Solid Fuels: Assessing the Environmental Burden of Disease at National and Local Levels. http://apps.who.int/iris/bitstream/handle/10665/42885/9241591358.pdf?sequence=1&isAllowed=y.

[6] Mishra V.K., Retherford R.D., Smith K.R. (1999). Biomass cooking fuels and prevalence of tuberculosis in India. Int. J. Infect. Dis..

[7] Po J., Fitzgerald J., Carlsten C. (2011). Respiratory disease associated with solid biomass fuel exposure in rural women and children: Systematic review and meta-analysis. Thorax.

[8] McCracken J.P., Smith K.R., Diaz A., Mittleman M.A., Schwartz J. (2007). Chimney stove intervention to reduce long-term wood smoke exposure lowers blood pressure among Guatemalan women. Environ. Health Perspect..

[9] Saha A., Kulkarni P.K., Shah A., Patel M., Saiyed H.N. (2005). Ocular morbidity and fuel use: An experience from India. Occup. Environ. Med..

[10] Zodpey S.P., Ughade S.N. (1999). Exposure to cheaper cooking fuels and risk of age-related cataract in women. Ind. J. Occup. Environ. Med..

[11] Sram R.J., Binkova B., Dejemek J., Bobak M. (2005). Ambient air pollution and pregnancy outcomes: A review of the literature. Environ. Health Perspect..

[12] Mishra V., Retherford R.D. (2007). Does biomass fuel smoke contribute to anaemia and stunting in early childhood?. Int. J. Infect. Dis..

[13] Smith K.R., Samet J.M., Romieu I., Bruce N. (2000). Indoor air pollution in developing countries and acute lower respiratory infections in children. Thorax.

[14] Ministry of Environmental Protection of China (2013). Report of Environmental Related Human Activity Patterns Survey of Chinese Population (Adults: 2013).

[15] World Health Organization (WHO) Indoor Air Quality Guideline: Household Fuel Combustion Review 8: Household Coal Combustion: Unique Features of Exposure to Intrinsic Toxicants and Health Effects. http://www.who.int/indoorair/guidelines/hhfc/Review_8.pdf?ua=1.

[16] World Health Organization (WHO) Guidelines for Indoor Air Quality: Household Fuel Combustion. http://www.who.int/airpollution/guidelines/household-fuel-combustion/en/.

[17] Kleeman M.J., Schauer J.J., Cass G.R. (1999). Size and composition distribution of fine particulate matter emitted from wood burning, meat charbroiling and cigarettes. Environ. Sci Technol..

[18] Streets D.G., Gupta S., Waldhoff S.T., Wang M.Q., Bond T.C., Bo Y.Y. (2001). Black carbon emissions in China. Atmos. Environ..

[19] Florig H.K. (1997). China’s air pollution risks. Environ. Sci. Technol..

[20] Tsai S.M., Zhang J.J., Smith K.R., Ma Y., Rasmussen R.A., Khalil M.A. (2003). Characterization of non-methane hydrocarbons emitted from various cooking stoves used in China. Environ. Sci. Technol..

[21] Dai S., Ren D., Chou C.-L., Finkelman R.B., Seredin V.V., Zhou Y. (2012). Geochemistry of trace elements in Chinese coals: A review of abundances, genetic types, impacts on human health, and industrial utilization. Int. J. Coal Geol..

[22] Kwong C.W., Chao C.Y.H. (2010). Fly-ash products from biomass co-combustion for VOC control. Bioresour. Technol..

[23] Streets D.G., Lu Z., Levin L., Ter Schure A.F.H., Sunderland E.M. (2018). Historical releases of mercury to air, land and water from coal combustion. Sci. Total Environ..

[24] Musmarra D., Karatza D., Lancia A., Prisciandaro M., Di Celso G.M. (2013). Adsorption of Mercury Chloride onto activated carbon on a new pilot scale plant. Chem. Eng. Trans..

[25] Karatza D., Lancia A., Musmarra D. (1998). Fly ash capture of Mercuric chloride vapors from exhaust combustion gas. Environ. Sci. Technol..

[26] Park D., Barabad M.L., Lee G., Kwon S.B., Cho Y., Lee D., Cho K., Lee K. (2013). Emission characteristics of particulate matter and volatile organic compounds in cow dung combustion. Environ. Sci. Technol..

[27] Kamata T., Reichert J., Tsevegmid T., Kim Y., Sedgewick B. Managing Urban Expansion in Mongolia. https://openknowledge.worldbank.org/bitstream/handle/10986/2464/550280PUB0Urba100Box34943B01PUBLIC1.pdf?sequence=1.

[28] Zhang J., Smith K.R., Ma Y., Ye S., Jiang F., Qi W., Liu P., Khalil M.A.K., Rasmussen R.A., Thorneloe S.A. (2000). Greenhouse gases and other airborne pollutants from household stoves in China: A database for emission factors. Atm. Environ..

[29] Hays M.D., Geron C.D., Linna K.J., Smith N.D. (2002). Speciation of gas-phase and fine particle emissions from burning of foliar fuels. Environ. Sci. Technol..

[30] Chen Y., Sheng G., Bi X., Feng Y., Mai B., Fu J. (2005). Emission factors for carbonaceous particles and polycyclic aromatic hydrocarbons from residential coal combustion in China. Environ. Sci. Technol..

[31] Fernández-Martínez G., López-Mahía P., Muniategui-Lorenzo S., Prada-Rodríguez D. (2000). Determination of volatile organic compounds in coal, fly ash and slag samples by direct thermal desorption/GC/MS. Analusis.

[32] Shah A.D., Huggins F.E., Shah N., Huffman G.P. The Form of Occurrence of Basic Elements in Coal and Their Behavior during Combustion. https://web.anl.gov/PCS/acsfuel/preprint%20archive/Files/353WASHINGTON%20DC08-900653.pdf..

[33] Catalfamo P., Pasquale S.D., Corigliano F., Mavilia L. (1997). Influence of the calcium content on the coal fly ash features in some innovative applications. Resour. Conserv. Recycl..

[34] Shah A.D., Huffman G.P., Huggins F.E., Shah N. Role of Calcium during Coal Combustion Revisited. https://web.anl.gov/PCS/acsfuel/preprint%20archive/Files/38_4_CHICAGO_08-93_1210.pdf.

[35] U.S. Environmental Protection Agency (EPA) (1990). Clean Air Amendments of 1990. 1st Congress (1989–1990).

[36] Pellerin C., Booker S.M. (2000). Reflections on hexavalent chromium. Health hazards of an industrial heavyweight. Environ. Health Perspect..

[37] Watson J.G., Chow J.C., Houck K.E. (2001). PM_2.5_ chemical source profiles for vehicle exhaust, vegetative burning, geological material, and coal burning in Northwestern Colorado during 1995. Chemosphere.

